# Deregulated expression of the HSP40 family members Auxilin-1 and -2 is indicative of proteostasis imbalance and predicts patient outcome in Ph^+^ leukemia

**DOI:** 10.1186/s40164-016-0034-5

**Published:** 2016-02-09

**Authors:** Margherita Vieri, Huimin Geng, John B. Patterson, Jens Panse, Stefan Wilop, Afshin Samali, Eric Chevet, Behzad Kharabi Masouleh

**Affiliations:** 1 Department of Hematology, Oncology, Hemostaseology and Stem Cell Transplantation, Medical Faculty, RWTH Aachen University, Aachen, Germany; 2 Department of Laboratory Medicine, University of California San Francisco, San Francisco, CA 94143 USA; 3 MannKind Corporation, Valencia, CA USA; 4 Apoptosis Research Centre, National University of Ireland, Galway, Ireland; 5 Department of Biochemistry, National University of Ireland, Galway, Ireland; 6 INSERM ERL440, Oncogenesis, Stress and Signaling, Université Rennes 1, Rennes, France; 7 Centre de Lutte Contre Le Cancer Eugène Marquis, Rennes, France

**Keywords:** Unfolded protein response, Acute lymphoblastic leukemia, Chronic myeloid leukemia, HSP40, DNAJ, Auxilin-1, Auxilin-2

## Abstract

**Background:**

Proteostasis is defined by the orchestrated control of anabolic and catabolic protein pathways. Disruption of proteostasis results in cell stress and adaptation to proteostasis imbalance is mediated by adaptive pathways such as the Heat Shock Response (including heat-shock proteins) or the unfolded protein response (UPR). The BCR-ABL1 kinase (Philadelphia chromosome) is the hallmark of chronic myeloid leukemia (CML) and defines a historically poor subset in acute lymphoblastic leukemia (Ph^+^ ALL). We previously demonstrated the importance of the UPR and particularly of the IRE1/XBP1 signaling axis in Ph^+^ ALL, while others demonstrated the therapeutic relevance of HSP70 in ALL. In this regard, HSP70 is regulated by smaller HSP40 s, whose function is so far poorly characterized.

**Results:**

Herein, we characterize the expression of HSP40 s in Ph^+^ ALL and CML. We show that these genes are not regulated in a pan-class manner and identify a homologous gene pair, namely Auxilin-1 (DNAJC6) and Auxilin-2 (GAK) with a unique expression profile. Overexpression of Auxilin-2, the ubiquitously expressed homologue of Auxilin-1 correlated with superior clinical outcome in ALL and was tightly linked to both IRE1 RNase and BCR-ABL1 kinase activities.

**Conclusions:**

Our findings suggest that HSP40 gens are uniquely regulated and provide a rationale for further studies between BCR-ABL1/IRE1-based therapies in combination with HSP40 inhibitors, thus opening potentially novel therapeutic avenues.

**Electronic supplementary material:**

The online version of this article (doi:10.1186/s40164-016-0034-5) contains supplementary material, which is available to authorized users.

## Findings

Control of protein homeostasis (proteostasis) is an essential driver of cell survival. In certain hematological malignancies such as multiple myeloma, the concept of disrupting proteostasis by weakening cancer cells’ adaptation to oncogene-induced increased metabolic demand and/or to challenging microenvironment has shown clinical success [1]. Under challenging conditions, proteostasis maintenance is achieved through the activation of selective adaptive machineries such as the heat shock response (HSR) or the unfolded protein response (UPR) [2, 3]. The HSR is mainly mediated by Heat Shock Factor-1 (HSF1) which is responsible for the induction of the heat shock proteins (HSPs), that were initially classified based on their molecular weight [4, 5] and are generally regulated by proteotoxic stress [6, 7]. For instance, the HSP40 (DNAJ) family of proteins is important for protein translation, folding, translocation and degradation, mainly by modulating HSP70 ATPase activity. While the role of HSP70 is well characterized, that of HSP40 proteins, specifically in hematological malignancies remains unclear [8]. Moreover, we have recently identified the IRE1/XBP1 axis of the UPR as an important survival cue in B-ALL [2, 9]. The IRE1/XBP1 axis is known to control the expression of several HSP40 family members [10], which suggests potential functional links between HSR and UPR.

**Fig. 1:**
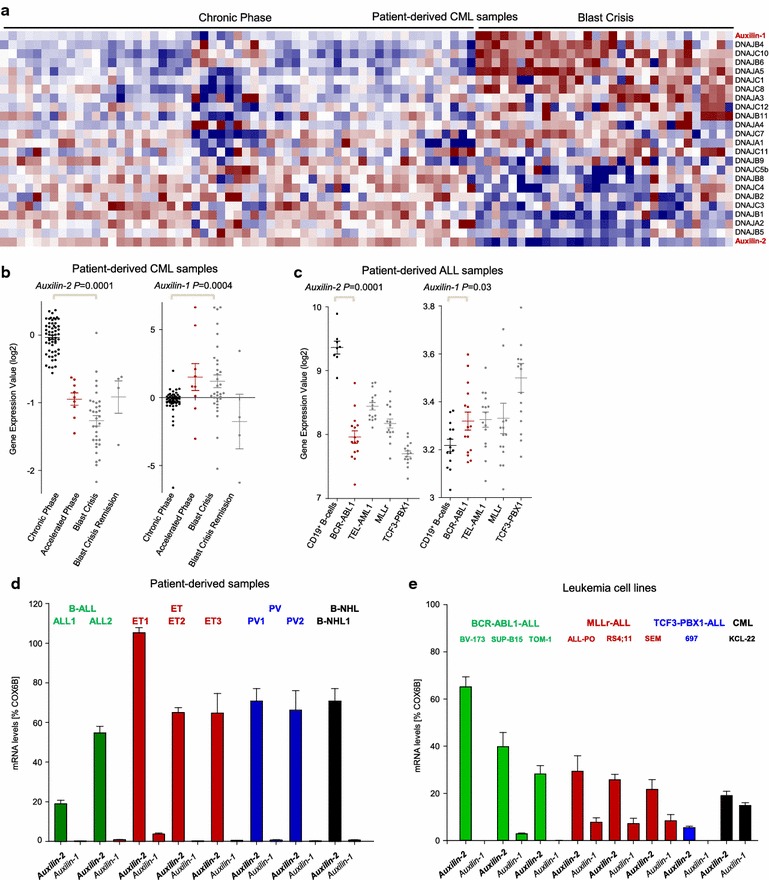
Heat shock protein members are differentially expressed in Ph^+^ leukemia. Gene expression profiling of CML patient samples in chronic phase and blast crisis. Highlighted are two homologues Auxilin-2 and Auxilin-1 (GEO accession number GSE4170) (**a**). In the same microarray (GEO accession number GSE4170) showed in Fig. 1a, CML patients were separated depending on their disease stage and the gene expression of Auxilin-1 and Auxilin-2 are shown after log2 transformation (**b**). The expression of Auxilin-1 and Auxilin-2 is shown for different ALL subsets carrying either BCR-ABL1, TEL-AML1, MLLr or TCF3-PBX1 lesions compared to CD19^+^ B-cells from healthy donors (http://www.stjuderesearch.org/site/data/ALL3/) (**c**). The mRNA levels of Auxilin-1 and Auxilin-2 were measured by qRT-PCR in primary cases of different hematological malignancies including ALL, Essential Thrombocythemia (ET), Polycythemia Vera (PV) compared to B-cell Non-Hodgkin Lymphoma (B-NHL) (**d**). Similarly, the mRNA levels of Auxilin-1 and Auxilin-2 were measured by qRT-PCR (used primers are shown in Table S2) in ALL and CML cell lines with different oncogenic lesions (**e**)

To test the potential interplay between those two adaptive pathways in leukemia, we studied the expression of 24 HSP40 members in Ph^+^ ALL and CML. When comparing gene expression of CML patients in chronic phase (CP) with terminal blast crisis (BC), HSP40 family members showed differential expression. Among the studied genes, two members, Auxilin-1 and -2 showed a rather unexpected expression profile. Whereas Auxilin-1 was highly expressed in BC-CML patients, Auxilin-2 expression was high in CP-CML and low in BC-CML patients (Fig. 1a). In addition, the expression of Auxilins in CML patients in all three phases (CP-CML, accelerated phase, BC-CML and after remission of BC-CML patients) revealed that Auxilin-2 decreased, while Auxilin-1 increased across disease progression of CML patients (Fig. 1b). To discriminate, if HSP40 expression profile was CML specific or related to the BCR-ABL1 kinase, we next studied the expression of the aforementioned genes in Ph^+^ ALL patient samples. HSP40 family members were similarly differentially expressed in Ph^+^ ALL patients compared to CD19^+^ B-cells from healthy donors (Additional file 1: Figure S1a). Interestingly in Ph^+^ ALL patients, the expression of Auxilin-1 was upregulated, while that of Auxilin-2 was again downregulated also in other ALL subsets when compared to CD19^+^ B-cells from healthy donors (Fig. 1c). We therefore focused on both Auxilins as they showed the most consistent and opposite expression pattern in ALL and CML patients (Additional file 1: Figure S1b).

Auxilin-1 is considered as the neuron-specific homologue of Auxilin-2 [11] with many overlapping functions, and genetic loss of Auxilin-1 causes upregulation of Auxilin-2 [11]. Since one caveat in gene-expression approaches is that a probe-set only detects changes between two conditions, even if the overall expression is marginal, we hypothesized that (a) while Auxilin-1 expression might be increased in comparison to pre-B cells, the overall expression might be negligible in baseline due to its neuron-specificity and (b) Auxilin-1 upregulation could cause a compensatory downregulation of Auxilin-2, thus biasing our studies. Our hypothesis was supported by the fact that mRNA levels of Auxilin-1 were low, while that of Auxilin-2 were detectable in primary cases of myeloproliferative neoplasms (MPNs), B-ALL and B cell Non-Hodgkin Lymphoma (B-NHL) and also established leukemia cell lines (Fig. 1d, e, Additional file 3: Table S1). These primary cases (Additional file 3: Table S1) were obtained in compliance with the institutional review board of the University of Aachen, ethical vote number EK 206/09. The potential upregulation of Auxilin-1 outside the nervous system is supported by its upregulation in hepatocellular carcinoma patients [12] leading to enhanced HCC proliferation and invasion, and suggesting neuron-independent and oncogenic functions. Following this notion, while Auxilin-1 expression was very low in baseline, treatment of Ph^+^ leukemia cell lines with the ER stresser Thapsigargin caused significant upregulation of both Auxilin-1 and -2 (Additional file 2: Figure S2c, d) suggesting that Auxilin-1 can be upregulated outside the nervous system. A similar pattern of regulation was previously observed for instance for the oncogene BCL6 which is non-detectable at baseline but can strikingly be upregulated upon Imatinib (IM) treatment emitting its oncogenic function [13, 14] by allowing leukemia cells to escape TKI-mediated cell death.

**Fig. 2:**
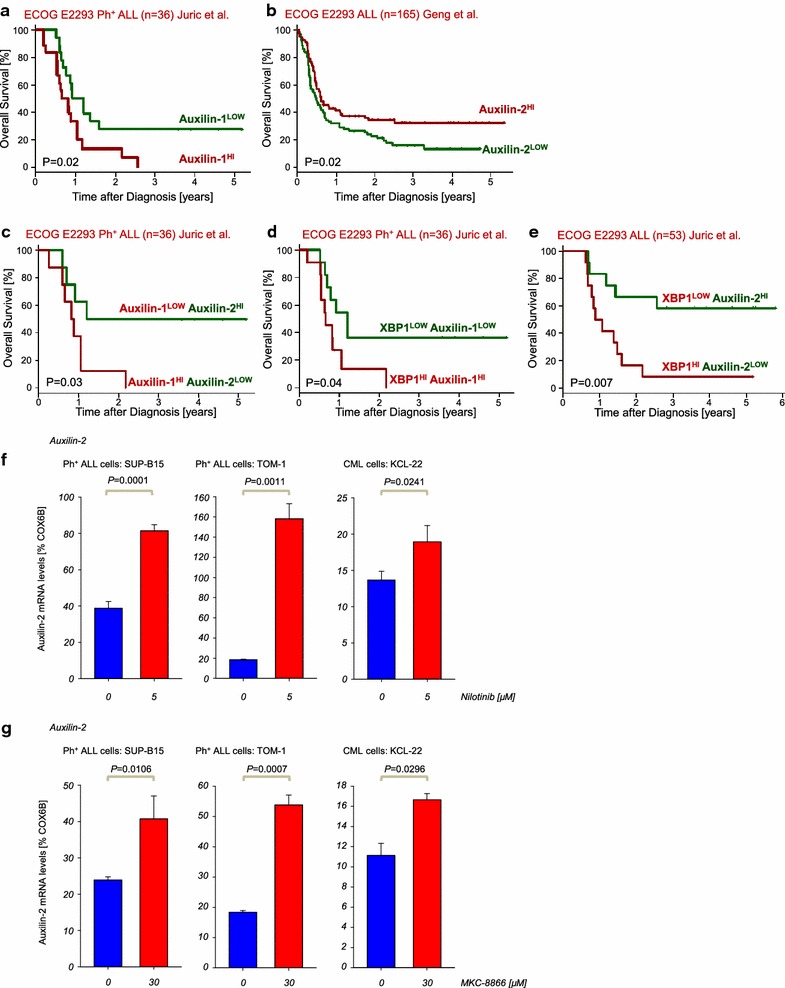
Auxillin-1 and 2 correlate with clinical outcome of ALL patients. In an analysis, ALL patients (ECOG E2993 [15, 16], n = 36, logrank test P = 0.02) were segregated into two groups based on high or low mRNA levels in respect to the median mRNA value of the Auxilin-1 and -2 probeset and the overall survival (OS) was assessed (**a**, **b**). Similarly, in a multivariate analysis; the ALL patients were segregated in two groups, according on high or low mRNA levels of Auxilin-1 and -2 (**c**), XBP1 and Auxilin-1 (**d**) or XBP1 and Auxilin-2 and overall survival was assessed (**e**). The patient subcohorts are mentioned in the according headlines (**a**–**d**). Auxilin-2 mRNA levels were measured by qRT-PCR in Ph^+^ ALL and CML cell lines (SUP-B15, TOM-1 and KCL-22) treated with or without the TKI Nilotinib for 16 h (5 µM) (n = 3) (**f**) and with or without the IRE1 RNase inhibitor MKC-8866 for 16 h (30 µM MKC-8866) (n = 3) (**g**)

We next studied the clinical relevance of both Auxilin genes. In two independent data sets of the ECOG E2993 clinical trial that included either 36 patients with Ph^+^ ALL [15], or 165 ALL cases [16], we segregated patients into two groups based on Auxilin-1 and -2 mRNA levels above or below their median expression value. ALL patients within the *Auxilin*-*1* ^*Hi*^ or *Auxilin*-*2* ^*Low*^ groups showed worse overall survival compared to those in the *Auxilin*-*1* ^*Low*^ or *Auxilin*-*2* ^*Hi*^ groups (*P* = 0.02; Fig. 2a, b). This supports the notion that Auxilin expression could be used as potential predictor of clinical outcome. A multivariate analysis based on the median expression of both genes with each other or with XBP1, which we had previously shown to be a predictor of poor outcome [9], revealed that under all conditions, when patients were segregated in the *Auxilin*-*2* ^*Hi*^, *Auxilin*-*1* ^*Low*^ (Fig. 2c) or *XBP1* ^*Low*^ groups (Fig. 2d, e), they had a better overall survival when compared to groups exhibiting *Auxilin*-*2* ^*Low*^, *Auxilin*-*1* ^*Hi*^ or *XBP1* ^*Hi*^ expression suggesting that the genes are predictors of survival.

The recent finding that Auxilin-2 knockout in MEFs does not cause the upregulation of Auxilin-1 suggests that the genes might act independently under certain conditions [17], even though Auxilin-1 knockout mice showed compensatory upregulation Auxilin-2 [11]. Taken together, the comparison of the studied HSP40 family members in leukemia suggested quite unique regulation (Additional file 1: Figure S1b). Since several HSP40 family members were upregulated in CML and Ph^+^ ALL, both driven by the BCR-ABL1 oncogene, we wanted to further understand if the expression of those genes might be linked to BCR-ABL1 kinase activity. We first studied their expression in a microarray of BCR-ABL1 transformed ALL cells treated with the tyrosine kinase inhibitor (TKI) Imatinib (Additional file 2 : Figure S2a). Several members which were previously downregulated in Ph^+^ ALL cases (Additional file 1: Figure S1a) were now upregulated upon TKI treatment including *Jdd1* (*Dnajc9*), *Mdg1* (*Dnajb9*), *Tpr2* (*Dnajc7*), *Erdj1* (*Dnajc1*), *Mcj* (*Dnajc15*) and *Auxilin*-*2* (*Gak*), while others, previously upregulated in Ph^+^ ALL cases such as *Auxilin*-*1* (*Dnajc6*), *Nedd7* (*Dnaja1*), *Rdj2* (*Dnaja2*)*, Tid1* (*Dnaja3*) and *Dnajc16* were downregulated upon TKI treatment (Additional file 1: Figure S2a). Due to the very low baseline expression of Auxilin-1 mRNA levels, we focused our studies on Auxilin-2 and verified the upregulation of Auxilin-2 mRNA levels in Ph^+^ ALL and CML cell lines treated with either Imatinib, or the 2nd generation TKI, Nilotinib (Fig. 2f, Additional file 2: Figure 2b). The differential regulation of individual HSP40 s upon TKI treatment suggest rather specific than a pan-class function. For instance NEDD7 (DNAJA1) which was downregulated by TKI-treatment has been described to be important for activation-induced cytidine deaminase (AID) function, a known oncogene in ALL [18, 19], whereas TKI treatment causes downregulation of AID [20]. Our finding might be the missing link between TKI treatment and AID downregulation through potential downregulation of NEDD7 (DNAJA1) and thereby negatively affecting AID function. Other important HSP40 members included MDG1 (DNAJB9) and TPR2 (DNAJC7), both upregulated by TKI-treatment and known to positively regulate the tumor suppressor p53 [21, 22]. We had previously shown that TKI treatment leads to downregulation of XBP1 s, while inhibition of XBP1 s activation by pharmacological inhibitors of IRE1 RNase activity caused apoptosis in Ph^+^ ALL cells [9]. As such the IRE1 RNase inhibitor MKC-8866 caused upregulation of *Auxilin*-*2* in Ph^+^ ALL and CML cells (Fig. 2g). This suggests that BCR-ABL1 kinase activity might cause potentially proteostasis imbalance leading to activation of the UPR, as previously identified by us and downregulation of individual HSP40 family members, which might hold the key to novel therapeutic approaches targeting the proteostatic network.

Taken together, our study provides for the first time, a more comprehensive overview of the expression and regulation of this understudied gene family in leukemias and more importantly shows that HSP40 family members can act as predictors of clinical outcome and are rather regulated by the BCR-ABL1 kinase activity specifically than in a pan-class manner.

## Additional files

**Additional file 1: Figure S1.** Heat shock protein members are differentially expressed in Ph^+^ leukemia. Gene expression profiling of Ph^+^ ALL patient samples compared to CD19^+^ B-cells from healthy donors (http://www.stjuderesearch.org/site/data/ALL3/) (**a**). A summary of the different expression profiles of the studied HSP40 (DNAJ) family members in this study is shown (**b**).

**Additional file 2: Figure S2.** Auxilin-2 expression is regulated by IRE-1 RNase and BCR-ABL1 kinase activity in Ph^+^ ALLGene expression profiling of BCR-ABL1-transformed ALL cells treated with Imatinib 2 μM for 24 h (GEO accession numbers GSE20987) (**a**). Auxilin-2 mRNA levels were measured by qRT-PCR in Ph^+^ ALL and CML cell lines (SUP-B15, TOM-1 and KCL-22) treated with or without Imatinib for 16 h (10 μM Imatinib) (n = 3) (**b**). Auxilin-2 (**c**) and Auxilin-1 (**d**) mRNA levels were measured by qRT -PCR in Ph^+^ ALL and CML cell lines (SUP-B15, TOM-1 and KCL-22) treated with or without ER stresser Thapsigargin for 16 h (100 ng/mL Thapsigargin for SUP-B15 and TOM-1 and 200 ng/mL for KCL-22).

**Additional file 3: Table S1.** Overview of patient-derived samples of Ph + ALL and cell lines. **Table S2.** Sequences of oligonucleotide primers used.
