# Supplementary material for: Deregulated expression of the HSP40 family members Auxilin-1 and -2 is indicative of proteostasis imbalance and predicts patient outcome in Ph+ leukemia
Source: Exp Hematol Oncol. 2016 Feb 9;5:5. doi: 10.1186/s40164-016-0034-5 (PMC4746784; doi:10.1186/s40164-016-0034-5)
Supplement: Supplementary file 1 — Additional file 1: Figure S1. Heat shock protein members are differentially expressed in Ph+ leukemia. Gene expression profiling of Ph+ ALL patient samples compared to CD19+ B-cells from healthy donors (http://www.stjuderesearch.org/site/data/ALL3/) (a). A summary of the different expression profiles of the studied HSP40 (DNAJ) family members in this study is shown (b). [file 40164_2016_34_MOESM1_ESM.pdf]

Supplemental Figure 1: *Heat shock protein members are differentially expressed in Ph⁺ leukemia*

a

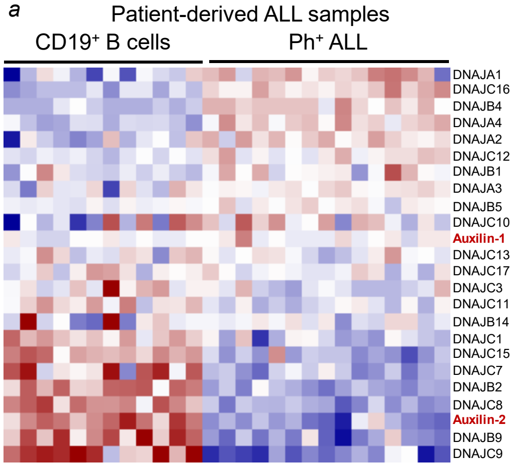

b

Gene	CML	ALL	Imatinib
DNAJC12	up	up	up
DNAJC11	up	down	
DNAJB6	up		
DNAJC4	down		up
DNAJC14			up
DNAJB5	down	up	up
DNAJC21			up
DNAJC30			
DNAJA3	up	up	
DNAJA4	up	up	up
**Auxilin-1**	up	up	down
DNAJB8	down		
DNAJC16		up	
DNAJC18			
DNAJD1			
DNAJB4	up	up	
DNAJC10	up	up	
DNAJC1	up	down	
DNAJC8	up	down	
DNAJA5	up		
DNAJB2	down	down	
DNAJB9	down	down	up
DNAJC3	down	down	
DNAJC5b	down		
DNAJC13		down	up
DNAJA1	up	up	
DNAJC7	up	down	up
DNAJB11	up		
DNAJA2	down	up	
DNAJB1	down	up	up
DNAJC9		down	up
DNAJB12			
DNAJC2			
DNAJC27			
**Auxilin-2**	down	down	up
