# Supplementary material for: Deregulated expression of the HSP40 family members Auxilin-1 and -2 is indicative of proteostasis imbalance and predicts patient outcome in Ph+ leukemia
Source: Exp Hematol Oncol. 2016 Feb 9;5:5. doi: 10.1186/s40164-016-0034-5 (PMC4746784; doi:10.1186/s40164-016-0034-5)

Supplemental Figure 2: Auxilin-2 expression is regulated by IRE-1 RNase and BCR-ABL1 kinase activity in Ph^+ ALL

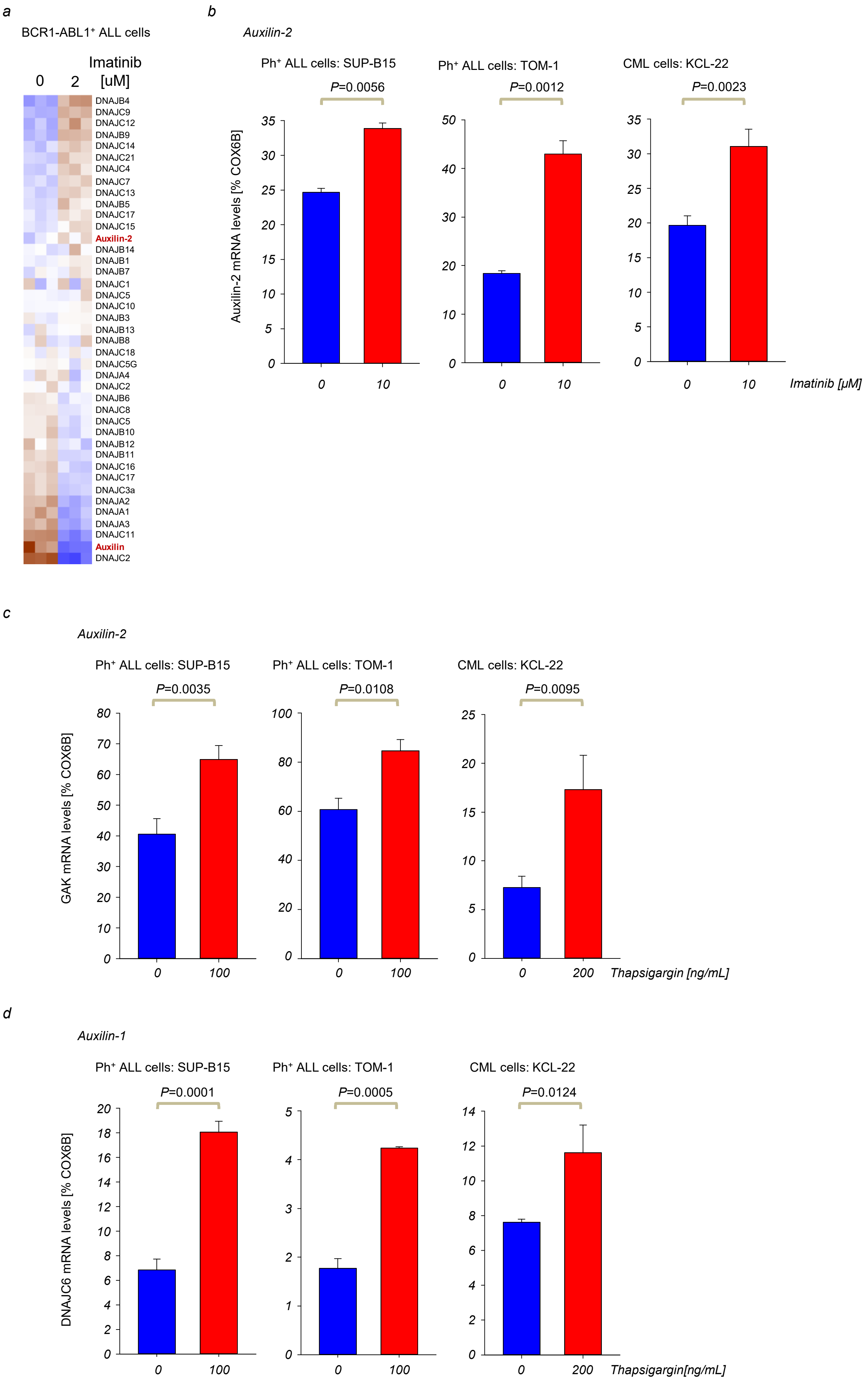

Supplement: Supplementary file 2 — Additional file 2: Figure S2. Auxilin-2 expression is regulated by IRE-1 RNase and BCR-ABL1 kinase activity in Ph+ ALLGene expression profiling of BCR-ABL1-transformed ALL cells treated with Imatinib 2 μM for 24 h (GEO accession numbers GSE20987) (a). Auxilin-2 mRNA levels were measured by qRT-PCR in Ph+ ALL and CML cell lines (SUP-B15, TOM-1 and KCL-22) treated with or without Imatinib for 16 h (10 μM Imatinib) (n = 3) (b). Auxilin-2 (c) and Auxilin-1 (d) mRNA levels were measured by qRT -PCR in Ph+ ALL and CML cell lines (SUP-B15, TOM-1 and KCL-22) treated with or without ER stresser Thapsigargin for 16 h (100 ng/mL Thapsigargin for SUP-B15 and TOM-1 and 200 ng/mL for KCL-22). [file 40164_2016_34_MOESM2_ESM.pdf]
