# Supplementary material for: Deregulated expression of the HSP40 family members Auxilin-1 and -2 is indicative of proteostasis imbalance and predicts patient outcome in Ph+ leukemia
Source: Exp Hematol Oncol. 2016 Feb 9;5:5. doi: 10.1186/s40164-016-0034-5 (PMC4746784; doi:10.1186/s40164-016-0034-5)
Supplement: Supplementary file 3 — Additional file 3: Table S1. Overview of patient-derived samples of Ph + ALL and cell lines. Table S2. Sequences of oligonucleotide primers used. [file 40164_2016_34_MOESM3_ESM.pdf]

Supplemental Table 1: *Overview of patient-derived samples of Ph^{+} ALL and cell lines*

Case	Karyotype	Clinical course	Gender/Age
**Primary cases**			
ALL1	46,XX,dic(9;20)(p11;q11),+20(13); 46,xx(8)	First Diagnosis	f/78y
ALL2	t(9;22)	First Diagnosis	m/72y
ET1	$JAK2^{V617F-}$, Calreticulin positive	First Diagnosis	m/28y
ET2	$JAK2^{V617F+}$, Calreticulin negative	First Diagnosis	m/57y
ET3	*ND*	ND	m/48y
PV1	$JAK2^{V617F+}$, Calreticulin negative, i45X, -Y [11]	First Diagnosis	m/74y
PV2	$JAK2^{V617F+}$, Calreticulin negative, 46, XY	First Diagnosis	nd/40y
B-NHL1	mantel cell lymphomas	Firs Diagnosis	m/77y
**Cell lines**			
BV-173	add(1)(q42), add(8)(p23), der(22)t(9;22)(q34;q11)	lymphoid blast crisis	m/45y
SUP-B15	t(1;1)(p11;q31), add(3)(q27), add(10)(q25), t(9;22)(q34;q11)	Relapse	m/9y
TOM-1	del(7)(p14),der(9)del(9)(q13q34) der(22)t(9;22)(q34;q11)	Refractory	f/54y
ALL-PO	t(4;11)	First Diagnosis	ND/4month
RS4;11	47/48<2n>X/XX, +8, +18, t(4;11)(q21;q23), i(7q)	Relapse	f/32y
SEM	45(40-46)<2n>XX, -13, t(4;11)(q21;q23), del(7)(p14)	Relapse	f/5y
697	46(45-48)<2n>XY, t(1;19)(q23;p13), del(6)(q21)	Relapse	m/12y
KCL-22	51(47-51)<2n>X, -X, +1, +6, +8, +8, +14, +22, del(1)(p22), t(9;22)(q34;q11), add(17)(p12-13), i(21q), der(22)t(9;22)(q34;q11)	blast crisis	f/32y

Supplemental Table 2: *Sequences of oligonucleotide primers used*

Quantitative RT-PCR

Human

COX6B_F	5'-AACTACAAGACCGCCCCTTT-3'
COX6B_R	5'-GCAGCCAGTTCAGATCTTCC-3'
GAK_F	5'-GGAGTACGACCAGCCGTATG-3'
GAK_R	5'-GGAGGTGTCCTTGAGGTTGG-3'
DNAJC6_F	5'- TGG GAT GCT AAA CCA GGA GG -3'
DNAJC6_R	5'- GGG TTT GCT GGC AAA GGA AG -3'
